# What every researcher should know about searching – clarified concepts, search advice, and an agenda to improve finding in academia

**DOI:** 10.1002/jrsm.1457

**Published:** 2020-10-08

**Authors:** Michael Gusenbauer, Neal R. Haddaway

**Affiliations:** ^1^ Department of Strategic Management, Marketing and Tourism University of Innsbruck Innsbruck Austria; ^2^ Chair for Strategy and Organization, Technical University of Munich Munich Germany; ^3^ Mercator Research Institute on Global Commons and Climate Change Berlin Germany; ^4^ Stockholm Environmental Institute Stockholm Sweden; ^5^ Africa Centre for Evidence University of Johannesburg Johannesburg South Africa

## Abstract

We researchers have taken searching for information for granted for far too long. The COVID‐19 pandemic shows us the boundaries of academic searching capabilities, both in terms of our know‐how and of the systems we have. With hundreds of studies published daily on COVID‐19, for example, we struggle to find, stay up‐to‐date, and synthesize information—all hampering evidence‐informed decision making. This COVID‐19 *information crisis* is indicative of the broader problem of information overloaded academic research. To improve our finding capabilities, we urgently need to improve how we search and the systems we use.

We respond to Klopfenstein and Dampier (*Res Syn Meth*. 2020) who commented on our 2020 paper and proposed a way of improving PubMed's and Google Scholar's search functionalities. Our response puts their commentary in a larger frame and suggests how we can improve academic searching altogether. We urge that researchers need to understand that search skills require dedicated education and training. Better and more efficient searching requires an initial understanding of the different goals that define the way searching needs to be conducted. We explain the main types of searching that we academics routinely engage in; distinguishing lookup, exploratory, and systematic searching. These three types must be conducted using different search methods (heuristics) and using search systems with specific capabilities. To improve academic searching, we introduce the “Search Triangle” model emphasizing the importance of matching goals, heuristics, and systems. Further, we suggest an urgently needed agenda toward search literacy as the norm in academic research and fit‐for‐purpose search systems.

**HIGHLIGHTS**

**What is already known?**

- To stay up‐to‐date, we researchers would need to read hundreds of research papers a day(!). Particularly, the avalanche of COVID‐19 papers exemplifies how we are chronically information overloaded.
- Evidence synthesis is more important than ever, yet we lack the knowledge and systems to effectively and efficiently identify the evidence bases for systematic reviews.

**What is new?**

- We claim that research discovery needs an urgent overhaul. Only with awareness of the basic concepts of academic searching, we can know how to make our search routines and systems fit‐for‐purpose.
- Our commentary clarifies these search concepts to point out the particularities of lookup, exploratory, and systematic searching. The “Search Triangle” model emphasizes that efficient and effective search only works when goals, systems, and heuristics are well matched.

**Potential impact for RSM readers outside the authors' field**

- Awareness for the importance of search literacy and search education is needed across disciplines.
- Better search skills not only help in research, but anywhere online.

We thank Klopfenstein and Dampier^1^ for their comment on our paper and for acknowledging the need to improve both PubMed and Google Scholar with functionalities that each is currently missing. We welcome increased scrutiny of the functionality of search systems and assessing whether these are truly fit‐for‐purpose as we struggle with information overload, particularly in times of crises like the current COVID‐19 pandemic. We are also very happy to see increased research attention on the systems that we use on a day‐to‐day basis for research discovery: functionalities that have remained unquestioned by those of us who are not information specialists for too long.

Indeed, we were overwhelmed by the substantial attention given to our paper^2^ (it currently has an Altmetric score of well above 300) and the positive comments we have received. This demonstrates the need for further scrutiny and improvement to academic search. It shows that researchers want to know more about the limitations of the systems they use to discover research, which limitations they must account for, and how to match their search strategies with each system. These decisions concerning the design of search strategies profoundly affect the resultant evidence that researchers identify, what they (often unknowingly) fail to identify, and what conclusions they draw based on the emergent evidence.^3^

In this article, we go beyond our original article and put the work of Klopfenstein and Dampier^1^ in a larger frame to discuss the kind of agenda setting needed to overhaul academic searching, and how this might be achieved by the research community.

## 1 SEARCHING AND BIAS IN TIME OF A GLOBAL CRISIS

The importance of effective and efficient identification of academic publications (hereafter referred to as *searching*) has become particularly evident in the current COVID‐19 pandemic: This pandemic is not only a medical crisis, but also an information crisis—not because there is no information on COVID‐19, but because there is more than we can handle. Recently, a Lancet editorial called this an “infodemic” and a “major threat to public health.”^4^ According to Semantic Scholar, more than 211 000 scientific articles exist to date on COVID‐19 across all disciplines^a^—almost all published in 2020. The National Institute of Health (NIH)'s *isearch COVID‐19 Portfolio*, an expert‐curated data collection, lists 60 297 medical COVID‐19 publications, whereas 79% were listed between May and August 2020^b^—amounting to an average daily(!) publication rate of almost 400 publications for medicine alone. This incredible *avalanche* of evidence is more than any individual can process. For any particular intervention (eg, mask‐wearing), one can find a confusing and conflicting set of studies purportedly demonstrating evidence for and against (eg, face masks for the public during the COVID‐19 crisis^5^). Thus, the way we can process and make sense of this overabundance of evidence is one of our greatest challenges the current infodemic shows us.

Currently there is overwhelming research attention trying to solve these information challenges in a diverse suite of innovative ways, each aiming to make COVID‐19‐related information readily discoverable and analyzable. On the one hand, there are dozens of new AI‐ or expert‐curated repositories: for example, *NIH LitCovid*, *NIH isearch*, *OPENICPSR COVID‐19 data repository*, *WHO COVID‐19 database (also linking to many other repositories)*, and *the Center for Disease Control and Prevention (CDC)* giving an overview of various repositories. On the other hand, there are new tools for visualization, access, categorization, and analysis of COVID‐19 information (eg, *SciSight* or *CoVis*), some of them via crowdsourced idea contests (eg, *Kaggle*) or hackathons organized by institutions around the globe. This host of new initiatives is important means to fight the *COVID‐19 infodemic* with improved information access and analysis. However, we argue that the information overload problem is exacerbated by the insufficient nature of the search systems we must use to find relevant information. If the systems and practices we have in place—to discover, analyze, and evaluate evidence—were fit‐for‐purpose, we would not need to battle COVID‐19 with context‐specific fixes that do only little in battling infodemics in all the other contexts. We advocate that fixing existing search systems and practices is at least as important as building new resources on top. This means raising researchers' awareness and understanding about the objectives of searching, along with improving search heuristics and the search systems that make the avalanche of evidence accessible. Klopfenstein and Dampier^1^ provide a good example of how best practices can be adopted across platforms and how researchers across disciplines can influence search system providers in how their systems should be improved.

One of the most critical factors that can easily limit the quality of our work is the belief that how we search academically is perfectly fine.^6^, ^7^ It is the belief that the systems we use on a daily basis and the habits we have developed throughout our careers are adequate to find effectively and efficiently. However, searching—one of the central elements of research work—needs trained skills, careful thought, and planning. We need to understand that where and how we search greatly impacts what we find and miss, what we conclude, and what we suggest for evidence‐informed decision making. Improving academic searching helps to improve the quality of science and helps fighting so‐called infodemics. Thus, much can be gained if we improve day‐to‐day academic searching for the millions of researchers worldwide.

We argue that the COVID‐19 pandemic is an important time to consider how to improve academic searching altogether. In this text, we clarify some important concepts of academic searching that are the subject of frequent misunderstanding, we introduce the “Search Triangle”—a user‐centric search model to understand the key characteristics of academic searching, and we explore why and how we need to overhaul academic searching to better inform decision making (Box 1).

Box 1 **How we expend much effort to get around a terrible searching environment**

COVID‐19 exemplifies an information crisis, with researchers building workarounds to cope with the insufficiencies of established search systems.

In theory, research on COVID‐19 could be readily identified by any user searching a database for “COVID‐19” and finding all relevant studies. However, several problems make this difficult, for example: (a) authors describe the concept using different terms; (b) many databases typically index records (and allow searches) based only on titles, abstracts, and keywords, missing potentially relevant terms in the full texts; (c) no single database catalogues all research; (d) poor search literacy in the research community means that errors or inefficiencies in searching are common; (e) paywalls restrict users' access to search facilities *and* the underlying research articles.

A suite of systems has been built to identify and assemble COVID‐19 relevant research to overcome these problems, making use of artificial intelligence (including machine learning), expert curation and screening for relevant information, and temporarily making resources Open Access.

These are admirable, but necessary only because accurate and efficient identification of (free‐to‐access) relevant research across comprehensive free‐to‐use databases does not exist.

## 2 UNDERSTANDING ACADEMIC SEARCHING—THE DIFFERENT SEARCH TYPES: LOOKUP, EXPLORATORY, SYSTEMATIC

As Klopfenstein and Dampier^1^ point out, Google Scholar is by far the most commonly used resource by researchers.^8^ This is not a coincidence—it allows straightforward, user‐friendly access to its vast database of research records.^9^ However, Google Scholar also shows us beautifully how a system can be perfectly suited for one type of search, while failing miserably for another. On the one hand it is very capable for targeted searches aimed at finding specific research articles,^10^ but has severe limitations in systematic searches (eg, a lack of transparency and reproducibility).^2^, ^11^ Most academics are unaware of the different types of searching that they use on a day‐to‐day basis.^12^ They use the systems they know and to which they are accustomed in ways for which they were never designed. The result is substantially biased, nontransparent, and irreproducible research studies. As researchers, we must start understanding the basic types of searching we engage in and how the objectives behind each search type (why we search) should determine the search methods—that is, system choice (where we search) and search heuristics (how we search).

There is much we can learn about searching from the information retrieval and information science literature: substantial efforts have been made to determine the types of searching at various level of granularity and the capabilities required by search systems. This discipline broadly distinguishes *lookup* and *exploratory searching* as the two key search types.^13^, ^14^ Lookup searches—also called “known item searches” or “navigational searches”—are conducted with a clear goal in mind and “yield precise results with minimal need for result set examination and item comparison.”^14^ ^(p. 42)^ Here, the search process should be swift and efficient so as not to disturb the user's workflow. However, lookup searches can also be used by researchers or decision‐makers for cherry picking. From the avalanche of studies, it is relatively easy to select evidence that supports a pre‐held belief or dogma that portrays a biased picture of reality. Sometimes, this cherry picking is deliberate; selecting whichever study provides support for an argument or decision that has already been made (ie, post hoc evidence use). And sometimes it is unintentional: when the first evidence encountered is assumed to be representative. In general, users want efficient and convenient information retrieval, particularly in lookup searches^15^, ^16^—the first result that fits typically satisfies the information need.^17^ However, as researchers or decision‐makers we should *explore* the available evidence in the least biased way or, better still, to additionally *search systematically* to have all available evidence for a specific topic (including the counter‐evidence to one cherry‐picked paper). Only then, we can be sure that our conclusions and decisions are sufficiently evidence‐informed.

As many topics are complex and require in‐depth understanding, and we cannot always trust anecdotal evidence (see lookup searches), we need *exploratory searches* to enrich our understanding. In exploratory searches, the search goal is somewhat abstract.^18^ It is a desire to better understand the nature of a topic, and the path to reaching this goal is not always apparent. Exploratory searching is a process characterized by learning^19^ where users aim to be exposed to a multitude of different, sometimes contradicting knowledge sources to build their mental models on a topic. Users “submit a tentative query to navigate proximal to relevant documents in the collection, then explore the environment to better understand how to exploit it, selectively seeking and passively obtaining cues about their next steps.”^20^ ^(p. 38)^ The heuristics that users employ and their ultimate goals change throughout the session as they make sense of the information, linking it to and adapting their mental models iteratively.^21^ A single search session might exclusively consist of lookup or exploratory searches, or might alter the two with mixed episodes of lookup (eg, fact checking, navigation) and exploratory searches (eg, discovery and learning). In exploratory searches, the search process often spans multiple sessions (ie, days, weeks, months) or media (eg, search, videos, offline conversations) where users engage with one or more systems, take notes, and save results to knowledge management systems. Users will often stop searching when they believe they have reached their goal (the information need is met) or when they conclude it cannot be reached with the resources available.^17^

While both lookup and exploratory searches are established concepts in information retrieval, they do not cover *systematic searches*—which we claimed in our paper^2^ is a distinct third search type with unique heuristics and requirements. Evidence synthesis, in the form of systematic reviews (including meta‐analyses) and systematic maps, has introduced many disciplines to the concept of *systematic searches*, with the goal to (a) identify all relevant records (within the resource constraints) in a (b) transparent and (c) reproducible manner.^2^ None of these three systematic search goals is shared by lookup or exploratory searches. Systematic searching is similar to lookup searching in that the search goal is known, yet the level of rigor in planning and reporting and the sophistication in the search scope are unmatched making it a distinct type of search activity. One key aspect of systematic searching is that the methods used to search should be a priori and developed through careful planning, ideally involving information retrieval experts.^22^

There are presently significant misunderstandings within the research community regarding what systematic searches should and should not entail. These misunderstandings have led to criticism of the systematic review method (compared to narrative reviews) which we find are unfounded—at least in view of the literature search phase that identifies the corpus of evidence for subsequent synthesis. A major criticism is that systematic reviews would not entail “hermeneutic circles” of iterative learning about a research concept, so that researchers would not include and reflect upon findings throughout the search process.^19^, ^23^ In practice, however, systematic searches should always be preceded by a thorough exploratory search phase, which in systematic reviews is called “scoping.” In this initial phase, the researchers use exploratory searches to familiarize themselves with the review topic: they extend their knowledge of concepts and language and define inclusion/exclusion criteria.^24^ Only then do they compose a systematic search strategy that aims to identify all available, relevant records on the topic in a transparent and reproducible manner (ie, well reported in the final manuscript). We agree that, when an initial scoping phase is missing, this may limit the validity of a systematic review greatly, since key terms and concepts may have been omitted or misunderstood, even by experts. Thus, for systematic reviews it is essential that *systematic searches* are preceded by a thorough *exploratory search* phase.

It is important to note that systematic searches *do not* themselves entail a learning process. They should be predefined, protocol‐driven, structured means of systematically searching, and extracting all potentially relevant bibliographic records. The search area is specified by these search steps (mostly through the use of *building blocks* and *snowballing* heuristics—see Table 1) and lays out all records for subsequent review of relevance/eligibility. In systematic searching, the “hermeneutic circle” of understanding should be well advanced (though it probably will never be finished). Thus, in systematic reviews using the building blocks heuristic (connecting concepts via Boolean operators) only the final iteration of the search string is truly systematic and must be transparently documented in detail. It is typically at this point that the researchers stop exploring for the purpose of improving the search area. While exploratory searches (*scoping*) might use the same heuristics (see Table 1), these initial searches are iterative and incrementally improve the search area used for the systematic review. Hence, one of the main advantages of systematic reviews is that they include both an exploratory and a systematic search, upon which the subsequent synthesis is based. Unlike in narrative reviews that often rely on exploratory searching alone, the systematic search phase in systematic reviews aims to maximize comprehensiveness and full transparency and reproducibility.

**TABLE 1:** Academic search types: Their goals, use cases, dominant heuristics, and key requirements to search systems

Search types	Goals	Use cases	Dominant heuristics (detailed information in reference)	Key requirements to search systems
Lookup^13^, ^14^, ^25^	To identify one or a small number of research articles that meet a narrow set of criteria. The search goal is clear for the user and the search path is simple. Users impatiently aim to fill their information gaps with quick, targeted searches	*Retrieval of specific facts* (well‐known knowledge need) *Question answering*, *verification* (also cherry‐picking) *Re‐finding searches* (search for something that was already identified before)	*Straightforward searches*, *navigation* ^17^ *Most specific first* ^26^ (search for a collectively exhaustive property)	*Efficient identification and retrieval (single session)*: Simple, straightforward search design (to not disturb the workflow in which lookup searches are embedded) High coverage for high recall/sensitivity (often via large, multidisciplinary systems) Effective interpretation by the system of what a user “means” when querying for specific records
Exploratory ^13^, ^14^, ^17^, ^25^, ^27^	To learn about a concept or body of research, including its characteristics (eg, terms, volume of evidence, type of research). Initially the search goal is fuzzy and ill defined, but gets clearer throughout the iterative search process. Depending on the extent of the cognitive gap between the identified information and what a user already knows, the process involves mixed feelings ranging from serendipitous joy to doubt and frustration	*General research discovery*, *learning*, *evaluation (incl*. *keeping up‐to‐date)* *Narrative reviews* *Scoping studies* (eg, in preparation for subsequent systematic reviews) *“Negative searches”* ^17^ (spotting of knowledge gaps: “no result” as a positive outcome)	*Wayfinding* ^17^ (learning with little prior knowledge) *Most specific first* ^26^ (search for a collectively exhaustive property) *Snowballing/Pearl growing* ^26^ (association) *(Post‐query) filtering* ^28^ (limitation based on meta‐information)	*Efficient navigation*; *learning support (multi‐session support)*: Low latency for fast navigation through connected space (for queries or browsing) Offering of various navigational options (querying, browsing, filtering) Offering of many different cues, so the user can quickly learn about a concept and make judgment calls of adequacy of search results (best‐match systems are typically favored over exact‐match systems^29^)
Systematic^2^	To identify all records on a specific topic through an unbiased, transparent, and reproducible search. The search goal is clear for the user after an initial exploratory phase (scoping). Users conduct a set of transparent and replicable search steps using complex search strings that have been carefully constructed to balance recall/sensitivity and precision,^30^ or other non‐query‐based heuristics (eg, snowballing, handsearching) in a systematic manner. Multiple bibliometric databases are searched to increase sensitivity	*Systematic reviews* *Meta‐analyses* *Systematic mapping* ^31^ *Bibliometric analyses*	*Building blocks* ^26^ (via Boolean operators) *Snowballing/Pearl growing* ^26^ (association) *Handsearching* ^28^ (systematic, manual screening) *Successive fraction* ^26^ (limitation based on exclusion list) *(Post‐query) filtering* ^28^ (limitation based on meta‐information)	*Comprehensive*, *transparent*, *reproducible*, *unbiased search area*; *efficient retrieval*: High precision (combination of high recall/sensitivity and high precision is often achieved by searching multiple specialized systems) Reproducible searches (repeated, identical queries retrieve the same results) Exact‐match systems that are transparent and support complex heuristics (eg, building blocks, Boolean) Access to/download of entire dataset

To date, systematic searching and its unique requirements have not been described by the information science literature. The influential work of Marchionini^14^ that distinguishes between lookup and exploratory searching lists synthesis work as part of exploratory search and fails to capture the nature of systematic searches (as employed in systematic reviews). To help distinguishing the three search types, we define and summarize them and add associated use cases and heuristics in Table 1.

## 3 CONDUCTING ACADEMIC SEARCHING—THE “SEARCH TRIANGLE”

**FIGURE 1:**
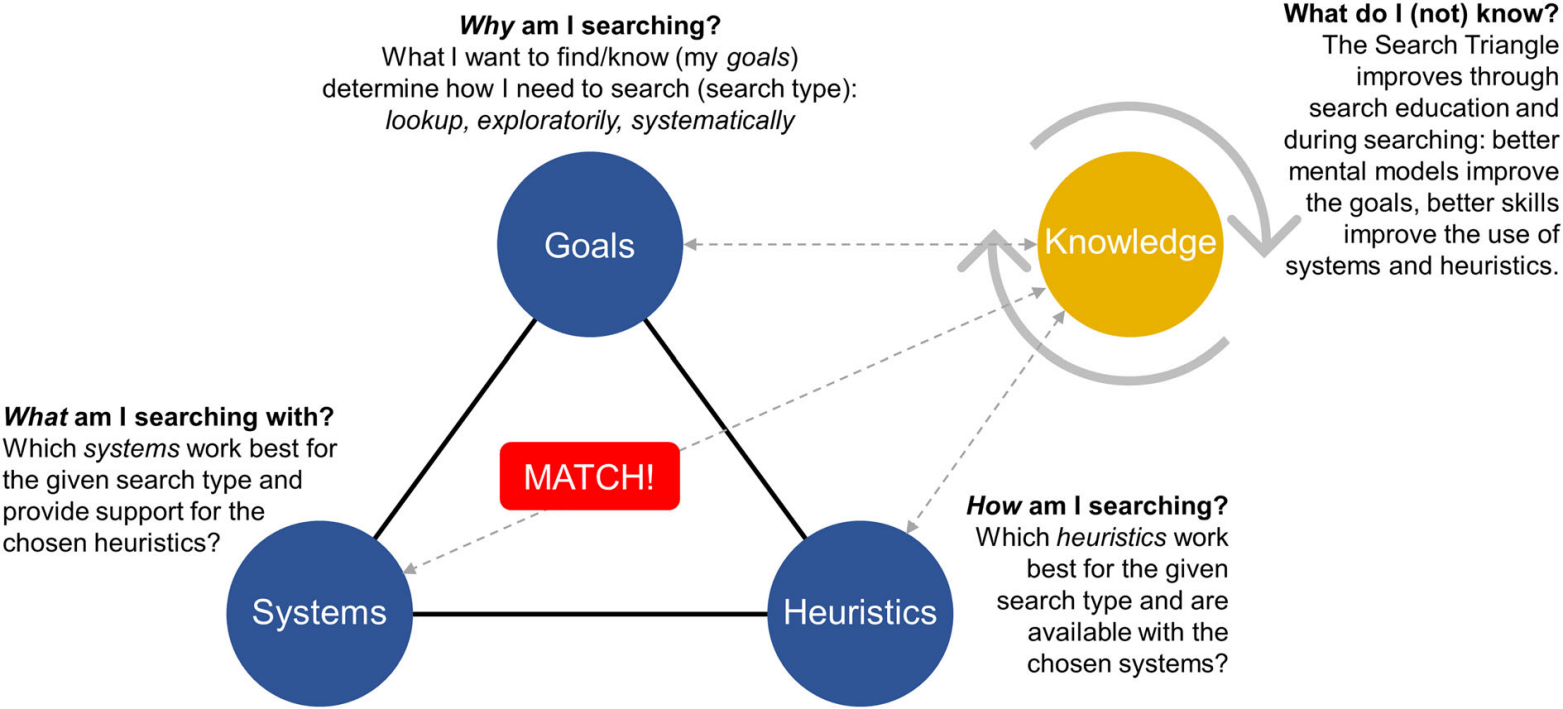
The “Search Triangle”: efficient and effective search only works when all three (search goals, search systems, and search heuristics) are matched well [Colour figure can be viewed at wileyonlinelibrary.com]

We contend that good academic searching starts with users thoughtfully establishing what their search goals are: that is, what they want to know/find. Given their search goals, search‐literate users know which type of search they need to engage in and can thus then select appropriate *heuristics* and *search systems*. Whether users are search literate, that is, are able to optimally match heuristics and search systems to their (evolving) search goals, determines the effectiveness and efficiency of finding and learning. We maintain that researchers—and indeed all information seekers—should understand the following three points that span a “Search Triangle” (see Figure 1):

1. The *users*' *goals*: what *needs* to be accomplished with the search task? For *lookup* searches, the goal is rapid and efficient identification of an artifact where the search area is already well known to users; for *exploratory* searches, the goal is learning about one or multiple concepts or about an evidence base; for *systematic* searches, the goal is the identification and extraction of all available records on an already well understood (scoped) topic.
2. The appropriate *heuristics*: *how* can the search be best conducted? The user must ask which (set of) heuristics best attain the search goal. While simple lookup searches come relatively intuitively with user‐friendly search systems like Google Scholar,^17^ the users' considerations of appropriate heuristics become important for effective explorative searches and particularly for systematic searches. Some of the most popular search heuristics described in information science literature (see Table 1) are *most specific first*, *wayfinding*, *snowballing (or citation chasing/chaining*, *pearl growing)*, *(post‐query) filtering*, *successive fraction*, *building blocks (*via *Boolean operators)*, or *handsearching*.^2^, ^17^, ^25^, ^26^, ^32^ It is important to note that no single heuristic is associated with a single search type. Rather, the choice of appropriate heuristics depends on the particular nature of the search goal and the options at hand, given a particular search system. For example, while building blocks are primarily used in systematic searching, they might also be used in particular types or phases of exploratory searching. Snowballing, for example, is used both in exploratory and systematic searching—yet with a different level of attention to rigor, transparency, and reproducibility.
3. The appropriate *systems*: which (set of) search system(s) best supports the required search type and the suitable search heuristics? It is important to know what *can* and *cannot* be accomplished, given the functional capabilities of a particular search system: eg, of the 28 systems analyzed in our paper^2^ only half can be recommended as stand‐alone systems in systematic searches. The selection of search systems, among the dozens available, defines what users will find. The search and retrieval capabilities are defined by the implicit characteristics of the search system in terms of functionality and coverage. It cannot be emphasized enough that no single search system is like the other and that each system is more or less adequate for specific search types (lookup/exploratory/systematic) in terms of coverage and supported heuristics.

## 4 IMPROVING ACADEMIC SEARCHING—SETTING AN AGENDA AND CALLS TO ACTION

To improve academic searching, we suggest an agenda that is rooted in three areas: (a) more awareness for the intricacies of academic searching; (b) better search education; and (c) pressure on search system providers to ensure their services are fit‐for‐purpose. We suggest key points that we believe the scholarly community must tackle, also jointly with institutions, publishing bodies, and search system providers.

### 4.1 More awareness for the intricacies of academic searching

Improving our search practice starts by creating awareness that *search literacy* is a crucial skill that does *not* come naturally through extensive computer and internet use, but needs to be trained in search education as part of research training.^33^, ^34^ Particularly, in the context of systematic reviews we must understand the two consecutive, yet distinct phases: exploratory searching and systematic searching. Too often, researchers skip the exploratory scoping phase and jump straight into systematic searching, while they still are (un/consciously) unsure about the meaning and language of central concepts.

*Search literacy* becomes increasingly needed as the number of search systems increases and the functionality they offer is diversified and continually updated, making them more or less (or not at all) suitable for specific search types. In recent years, we have seen the introduction of numerous new systems (eg, Microsoft Academic, Dimensions.ai, Meta, The Lens, Semantic Scholar) and techniques (eg, personalized or AI‐based search results) in academic search. Researchers must understand that these systems are all different and that system choice will heavily affect (or bias) what they will find. At the moment, the algorithms of so‐called semantic search systems (eg, Google Scholar or Semantic Scholar) and the precise methods of how they select and rank what is shown on the results page are unknown. However, there is evidence^6^, ^35^ that these opaque algorithmic decisions influence how we researchers conduct science—what we find, what we cite, how we argue, what we conclude. The academic community needs to be aware of these biases, and equip itself with the know‐how to avoid basing entire research projects (particularly systematic reviews) on potentially biased evidence bases (eg, Burivalova et al^36^).

We currently see an alarming absence of awareness for search system choice. This is evident in the many publications that confuse search system types^37^: foremost platforms used to access databases (such as Web of Science) and the databases themselves (such as Science Citations Index Expanded). These types are confused not only by research users more generally, but also by experts in the field of Scientometrics and others, where researchers specifically research these systems. This lack of awareness illustrates how urgently we need to start understanding academic search: the search types, the heuristics, and the search systems—to find more, faster, and with less bias.

*Call to action*: We must raise awareness across research communities—among students, educators, journal editors, university teaching boards, and interest organizations—of the intricacies of academic searching and how it can be improved. Organizations like the Collaboration for Environmental Evidence,^38^ Campbell Collaboration,^28^ and Cochrane^39^ can play important roles in creating awareness for the intricacies of academic search by updating their guidance to include more nuanced academic search advice. Additionally, academic journals must ensure that editors and peer‐reviewers are aware of the importance of robust search methods to encourage more rigor in academic searching (even more so as evidence synthesis become increasingly valued and prevalent). Only with this awareness, we can adequately link search goals to appropriate heuristics and systems to perform “good science”:

- *It starts with the users*' *goals*: Raising awareness so users understand what goals they want to reach with their searching and with which (implicit) scientific standards the specific search types (lookup/exploratory/systematic) are associated.
- *Search types*: Raising awareness that searching is *not* always a quick “just Google (Scholar) it,” but in fact can be described by a “Search Triangle” that needs a matching of search goals/types with heuristics and systems (see Figure 1).
- *Search heuristics*: Raising awareness that we could use better methods in searching databases and should be designing our searches around suitable heuristics that allow us meeting our diverse search goals.
- *Search systems*: Raising awareness that search systems are all different, not only in coverage, but also in the functions they offer and (equally important) they do *not* offer. It is also vital to understand that searches can be biased through the use of algorithms to adjust the order of records in search results.^40^ In the context of systematic reviews, ensuring transparent and adequate reporting of which systems are searched must be a key responsibility of research authors, editors, and peer‐reviewers. Systems to support reporting of this level of detail are available (eg, PRISMA‐S^41^) and should be adapted to all forms of research involving searching, not just systematic reviews.

### 4.2 Better search education: Toward search literacy as the norm

To build *search literacy* that enables quick choices of both heuristics and systems given an imminent information need involves more than the day‐to‐day search experience we researchers have at hand. Instead, it requires targeted *search education*. Such education has been shown to significantly improve search quality.^32^, ^42^ Without anchoring search education in research curricula, much scholarly search effort will remain wasted.^43^, ^44^

*Call to action*: we must make *search literacy* a priority in research education:

- *What needs to be taught?* Since many researchers think their current search practices and systems suffice, we need to raise awareness about problems associated with search illiteracy^45^ in combination with showing better ways of searching. The teaching objective should be to improve knowledge and skills on how to effectively and efficiently find, evaluate, manage, and use information. Taught concepts should include matching: (a) user goals/search types, (b) search heuristics, and (c) search systems. Among others, this includes awareness for the importance of adequate language to describe concepts, the ability to formulate comprehensive, yet precise search strings and the skills to search the most suitable systems.
- *Who teaches it?* University libraries can play a key role in making emergent and established researchers and professionals search literate.^46^ In times where fewer people visit physical libraries, more advice is required in the online realm. The freed‐up resources of librarians and information specialists might be used to teach new formats to students and scholars about search.
- *How can it be taught?* Search literacy can be taught as stand‐alone course or extend existing teaching concepts on digital literacy or information literacy, particularly also in courses on evidence‐based research.^47^, ^48^, ^49^ As many institutions lack libraries—particularly the ones from resource‐constrained environments—education should also be freely and easily accessible to all (ie, Open Education). Perhaps this could be organized most impactfully as self‐paced online training or freely licensed teaching materials that can be used and adapted by trainers across the world.

### 4.3 Toward fit‐for‐purpose search systems

No two‐search systems are identical, and none is perfect. The reason for the great popularity of some systems is *not* because of their adequacy for each of the three search types we describe,^2^ but rather because of their ease of use in day‐to‐day research practices. In the last decade, the tremendous success of Google Scholar has shown that users generally want to search intuitively, with as little effort as possible.^17^

In terms of functionality, two broad types of search systems exist at present: the traditional “comprehensive‐transparent” (eg, ProQuest, PubMed, Web of Science) and the newer “efficient‐slick” (eg, Google Scholar, Semantic Scholar). The first type allows users to specify their search to the greatest detail, while the second identifies relevant results quickly. The most popular systems are efficient‐slick, while it seems the traditional systems have focused on new features rather than low latency and accessibility. The mission statements of some popular and newly created semantic systems—including Microsoft Academic, Semantic Scholar, and Meta—can be summarized with: *simpler and more efficient searching*, *faster results*. Their aim is the fast satisfaction of researchers' information needs, without detours.

While this increase in search efficiency is generally positive, it comes at a cost. We see two fundamental problems: first, in these semantic search systems it is opaque algorithms that decide about the “right” information that is shown (either absolutely or by order). We currently have neither insight, nor control over these decisions. This is particularly problematic for systematic searching, where our study has shown that all semantic search systems in our sample failed to meet the requirements.^2^ Second, we must stay alert as these efficient‐slick systems aim at transforming ‘inefficient’ exploratory searching into ‘efficient’ lookup searching (eg, through presentation of pre‐selected cues). This means exploratory searching (and thus learning) might be more and more crippled toward quick, unconsciously biased lookup searching (cherry picking) that users more and more expect when engaging with online systems.^50^ To be innovative as an academic it is essential to build own mental models, to connect disconnected threads that have not been connected before—by neither machine nor human. If we reduce these “hermeneutic circles” for the sake of efficiency, we must be aware of the drawbacks. It clearly makes a difference if users are efficient in finding information on for example “the capital of Kiribati” or to which president to vote in the next election. While the first should be efficient (lookup), the latter should largely remain exploratory where users are presented with a balanced information diet. We must be careful and stay alert with systems that give us readymade answers. We must question the algorithms (AI, machine learning) and behavioral data that are used to create relevance rankings and thereby determine what researchers get to see and what not.^6^ Unfortunately, it seems as if the greatest level of effort of many search systems does not go into what researchers need to accomplish in *all* their search tasks, but rather in making users satisfied (and not smarter) sooner.

We researchers need the best of both worlds to ensure the best research outcomes: we need efficient‐slick and comprehensive‐transparent. We claim that, at present, systems could do much more in different areas than fine‐tuning for the sake of efficient lookup searching—particularly in the realm of evidence synthesis.

*Call to action*:

- *Greater transparency*: Search functionalities – that is, what can(not) be done with a search system (see our paper^2^ for details of how this can be quantified) need to become transparent. This can only be done through an independent assessment of the claims of search system providers—our study, for example, has shown that one out of four systems promoted the functioning of search options (ie, Boolean search) that we found was flawed.^2^ Additionally, we need clarity in the algorithms that semantic search systems use to fine‐tune their search results to reflect on how this impacts research work. With transparency, users can make informed choices on which systems to choose and systems can benchmark to compete for users, all driving a healthy competition toward better options of search facilities.
- *Toward fit‐for‐purpose—matching requirements with technical possibilities*: Some of the limitations we academics are confronted with when using search systems exist because of a lack of communication between the technical (what is possible) and the applied (what is needed). We believe the tools and features of search systems would greatly benefit from effective guidance and feedback from the research community (besides the user testing, etc. they are already doing). By establishing *clear rules* (similar to what systematic search needs to fulfill), we can help to direct the improvement of search systems, and thereby improving access to future‐proof search functionalities. Here we need to involve information technology research methods that have a long history in investigating the performance of particular search features or technologies (eg, reinforcement learning,^51^ interactive intent modeling,^52^ query expansion^53^). We do not need to reinvent the wheel, yet we need to improve communication between library science/evidence‐based research methodologists (the applied) and information technology research, and importantly: the search systems we use on a daily basis (the technical). Klopfenstein and Dampier^1^ demonstrate that: first, there is much room for improvement of search system workflows, features, and supported heuristics. Second, cross‐database integration might make sense to combine strengths of different databases (the coverage of Google Scholar and the specialized features of PubMed). Third, transparent comparison of features across search systems can be key to improve the systems we have. To improve our systems, we need an understanding of the exact requirements systems need to have for specific search types. The academic community should rally around these definitions and search types and demand clarity on which systems are best suited for which type of searching.
- *Organize change*: To see real improvements in academic searching, we must coordinate around the issue of fit‐for‐purpose research discovery. Without organized pressure this will remain a top‐down decision process, where search organizations continue deciding on what systems we use without hearing the requirements of the academic community. The popular example is Google Scholar that has refrained from improving transparency despite the many calls from, for example, the Scientometrics community in recent years.^9^, ^11^, ^54^, ^55^ COVID‐19 has shown us that positive change is possible if the pressure and a sense of urgency is great enough: for example, search systems and publishing houses have met criticism of impeding efficient, Open Science by temporarily making COVID‐19 literature Open Access.^56^ Thus, we need to decide how to organize the academic community to put pressure on search system providers to design their systems in such a way that supports the three different types of searching. Such demands for improvements are warranted and should be heard particularly by the systems we are (collectively) paying for through subscription fees. As a consequence, a great amount of effort (and thereby public money) could be saved if deliberately imposed barriers (such as view and download limits, paywall barriers, or data access restrictions) were to be removed and search functionalities improved.

## 5 CONCLUSION

The tremendous thirst for information on COVID‐19 by policy makers, managers, and the general public has triggered an avalanche of research. While this ever‐growing evidence base shows the academic system's capabilities to produce evidence rapidly and on tremendous scale, it has also triggered a COVID‐19 infodemic. The information overloaded researchers found across subjects and disciplines highlight the vital need to improve research discovery. Newly developed COVID‐19‐specific tools and repositories are certainly helpful, yet we also must carefully evaluate what these new technologies promise and why current systems are not already adequate. To fight the COVID‐19 infodemic—and in fact all infodemics—we argue it is essential to foremost fix how we search for scholarly evidence on a daily basis. This not only has the potential to improve search literacy across academic disciplines, but may also have spillover effects to a broader audience by educating students, organizations, and institutions.

Currently, we are at an exciting point in the development of informatics: an avalanche of research publications is being catalogued more comprehensively by an expanding suite of different bibliographic databases and research platforms (interesting developments include Dimensions.ai and The Lens). Intelligent research discovery systems make it easier than ever to identify research that is relevant to us.^9^ However, it has been shown how relevance rankings direct science, a phenomenon that is aggravated with new the technologies of artificial intelligence and machine learning that introduce black‐box relevance rankings and auto‐suggestions to the daily scientific enterprise of millions of scholars. Before we have fully understood the cost of such efficient systems, we need to be cautious for how we use them. Without full understanding of the different types of searching and their requirements, users of search systems are increasingly at risk of identifying a biased or unrepresentative set of search results.^6^ We must improve our understanding of the intricacies of searching and ensure search systems are specifically designed to tackle all modes of searching: only then can we conduct research with a more balanced information diet and make sure the evidence bases on which decisions are based are fit‐for‐purpose.

We currently see the greatest search issues in *systematic searching*: both in terms of the inadequate systems we have at hand and the uneducated researchers that use them. If the available search systems were specifically tailored to the needs of search‐literate researchers, the evidence we could produce would be of significantly greater validity and at significantly lower cost. Facilitating and thus accelerating the creation of systematic reviews could particularly help in times of crises—such as we experience today with COVID‐19.

We hope the clarification of academic search concepts, the advice in form of the “Search Triangle” model and our calls to action will help improving academic search. We hope our work informs decision making in academic searching and might prove useful in structuring and conducting search education toward search literacy as a methodical skill every academic exhibits and cherishes.

## CONFLICT OF INTEREST

The author reported no conflict of interest.

## Data Availability

Data sharing is not applicable to this article as no new data were created or analysed.

## Endnotes

^a^ Searching for “COVID‐19,” a suggested keyword by Semantic Scholar (https://www.semanticscholar.org/search?q=COVID-19&sort=year), accessed on 1 September 2020.

^b^
*Isearch* was accessed with a blank query to access all records on the database. (https://icite.od.nih.gov/covid19/search/#search:searchId=5f4dff240e329a34eac4e89f), 60 297 records as of 3 August 2020, 47 514 between 1 May and 31 August 2020, accessed on 1 September 2020.
